# Relationship between BMI and risk of impaired glucose tolerance and impaired fasting glucose in Chinese adults: a prospective study

**DOI:** 10.1186/s12889-022-14912-0

**Published:** 2023-01-03

**Authors:** Xin Zhang, Yankun Yue, Shaobo Liu, Xiangfeng Cong, Wenjuan Wang, Jianhong Li

**Affiliations:** 1 National Center for Chronic and Noncommunicable Disease Control and Prevention, Chinese Center for Diseases Control and Prevention, Beijing, 100050 China; 2 Fu Xing Hospital, Capital Medical University, Beijing, 100045 China

**Keywords:** Body mass index, Impaired glucose tolerance, Impaired fasting glucose, Prospective study

## Abstract

**Background:**

Current studies in most Western countries have largely focused on body mass index (BMI) as an important risk factor for impaired glucose tolerance (IGT) and impaired fasting glucose (IFG), which have different pathophysiological bases. In people with obesity, the prevalence of IGT is higher and the prevalence of IFG is lower. The prevalence of IGT in the Asian population is higher than that in the white population, and the obesity rate in China is still increasing. However, few cohort studies explore the relationship between BMI and the incidence of IGT and IFG in China. We aimed to explore the relationship between BMI and the risk of IGT and IFG in Chinese adults and analyze the differences between them.

**Methods:**

The baseline data were obtained from the 2010 China Chronic Disease and Risk Factor Surveillance, of which 20 surveillance sites were followed up from 2016 to 2017. Finally, in this study, a total of 5,578 studies were grouped into BMI categories of underweight (BMI < 18.5 kg/m^2^), normal weight (18.5–23.9 kg/m^2^), overweight (24.0–27.9 kg/m^2^), and obesity (≥ 28.0 kg/m^2^). We used the unconditional logistic regression model to analyze the relationship between BMI and the risk of IGT and IFG.

**Results:**

During an average follow-up of 6.4 years, 562 developed IGT and 257 developed IFG. After age, gender, urban and rural areas, physical activity, family history of diabetes, hypertension, abdominal obesity, dyslipidemia, and other factors were adjusted, overweight increased the risk of IGT by 35% [odds ratio (*OR*) 1.35, 95% confidence interval (*CI*) 1.08–1.70], and obesity increased the risk of IGT by 77% (*OR* 1.77, 95% *CI* 1.27–1.47). After the factors consistent with the above were adjusted, only obesity increased the risk of IFG by 122% (*OR* 2.22, 95% *CI* 1.39–3.54).

**Conclusions:**

In China, obesity is an important risk factor for IGT and IFG, and the risk of IGT increases during the overweight stage.

**Supplementary Information:**

The online version contains supplementary material available at 10.1186/s12889-022-14912-0.

## Introduction

Obesity is the energy imbalance between calorie intake and consumption, and it is one of the most obvious but neglected public health problems [1]. Both developed and developing countries are faced with the threat of obesity [2]. In China, the proportion of overweight adults increased from 32.5% in 2013 to 34.5% in 2018, and the proportion of obesity increased from 14.1% in 2013 to 16.5% in 2018 [3]. As is known, obesity is a risk factor for prediabetes and diabetes [4, 5]. According to the statistics of the International Diabetes Federation, in 2021, there were approximately 541 million impaired glucose tolerance (IGT) patients worldwide, and the number is expected to reach 222.7 million by 2030 and 730.3 million by 2045. The number of impaired fasting glucose (IFG) patients in the world was approximately 319 million in 2021 and will be 369.7 million by 2030 and 440.8 million by 2045 [6]. Weight control is widely promoted as a major preventive measure to reduce the burden of prediabetes and subsequent diabetes [7]. There are few cohort studies on the specific effects of body mass index (BMI) and the risk of IGT and IFG in China, and the different relationships between obesity and the risk of IGT and IFG have important public health significance for future screening and the development of corresponding interventions. Our study was based on the data from the 2010 China Chronic Disease and Risk Factor Surveillance, and a follow-up survey was conducted from 2016 to 2017. The cohort was used to analyze the relationship between adult BMI and the risk of IGT and IFG in China, to put forward targeted suggestions for the prevention and control of prediabetes and diabetes, and to provide data support for our government to formulate corresponding prevention and control policies.

## Methods

**Fig. 1:**
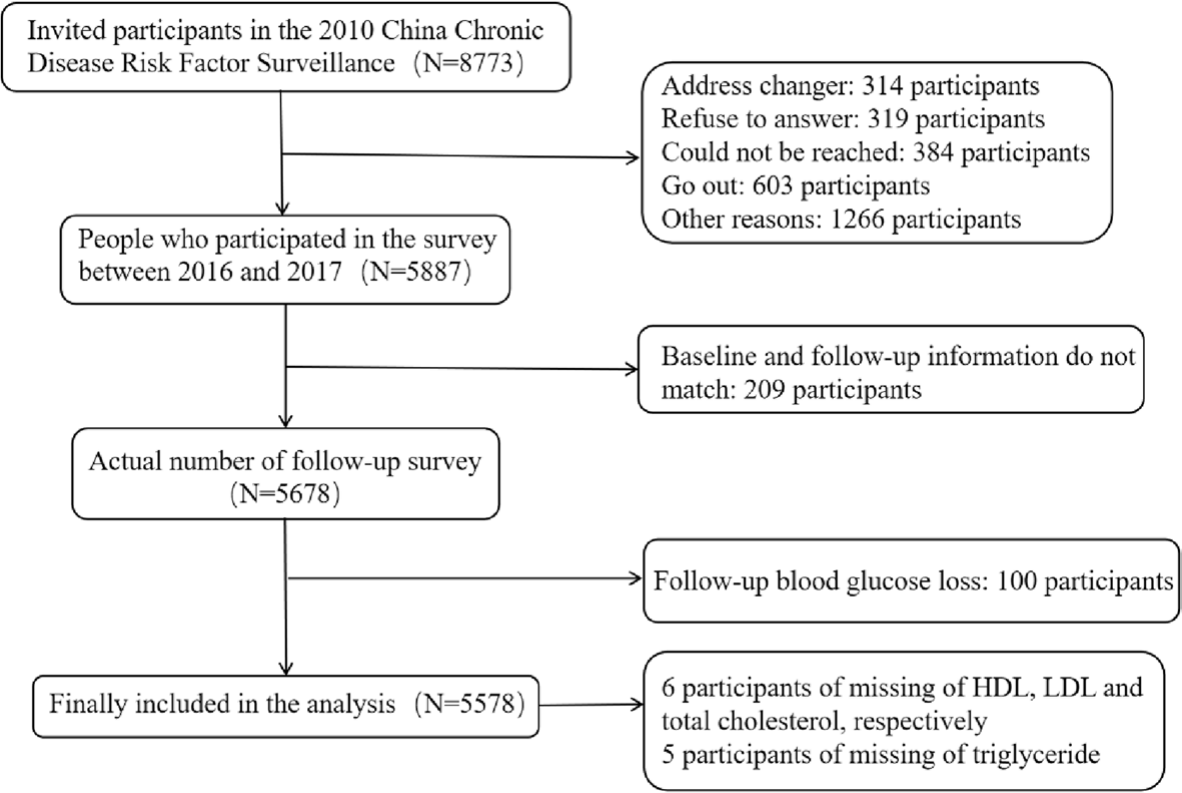
The flowchart for participants inclusion

The China Chronic Disease and Risk factor Surveillance (CCDRFS) is a series of nationally representative regular cross-sectional surveys among Chinese adults conducted every 3 years (every 5 years since 2018) since 2004. The content concerns the population’s chronic health status (such as hypertension, diabetes, stroke, dyslipidemia, cancer, myocardial infarction, and chronic obstructive pulmonary disease) and related risk factors (such as smoking, harmful drinking, lack of physical exercise, and dietary imbalance) [8]. In this study, 10 provinces (Hebei, Jilin, Heilongjiang, Zhejiang, Jiangxi, Henan, Hunan, Sichuan, Guizhou, and Shanxi) were selected from the 2010 CCDRFS. Two monitoring sites (one in an urban area and one in a rural area) were selected in each province. A total of 8,773 participants (excluding people with IGT, IFG, and diabetes and those younger than 18) were invited. A follow-up survey was conducted from 2016 to 2017, but 2,886 of the invited participants refused to participate. After 209 participants whose baseline and follow-up information did not match were removed, a final total of 5,678 participants completed the follow-up. After the exclusion of 100 participants with follow-up plasma glucose deficiency, 5,578 were included in the analysis. Among them, the missing data account for only 2% of the total sample (Fig. 1). In 2010, the CCDRFS was performed in 162 surveillance sites across the country (including the Second Agricultural Division of Xinjiang Production and Construction Corps). The representative samples were selected by using multistage stratified cluster sampling, and the residents aged 18 and older in the sample area were investigated. With the use of the sampling method proportional to the scale, four townships (streets and regiments) were randomly selected from each monitoring point, three administrative villages (neighborhood committees, and companies) were randomly selected from each sample township (streets and regiments), and one resident group (approximately 50 households) was randomly selected from each village. Finally, one person was randomly selected from each household by using the Kish table method. The details of this project can be found in previously published literature [9]. This study has passed the review of the Ethics Review Committee of the National Center for Chronic and Noncommunicable Disease Control and Prevention, Chinese Center for Disease Control and Prevention (approval number 201524B). All methods were performed following the tenets of the Declaration of Helsinki. All the participants signed the informed consent form, and for the illiterate participants, the informed consent of their parents and/or legal guardians was obtained.

Both baseline and follow-up surveys included an inquiry survey, physical measurements, and laboratory testing. The inquiry survey was conducted in the form of face-to-face interviews. The questionnaire included basic personal information, lifestyle (including smoking, drinking, diet, and physical activity), occurrence, diagnosis, and control of chronic diseases. Body measurements included height, weight, waistline, and blood pressure. The laboratory test required each participant to collect three tubes of 5–6 mL of venous blood after fasting for 10 h and another tube of 1 mL of venous blood after taking 75 g of glucose for 2 h. The indexes included fasting plasma glucose, 2-h plasma glucose after glucose load, glycosylated hemoglobin, and blood lipid. For detailed methods, please refer to the 2010 CCDRFS [9] and other literature reports [10].

### Physical measurements

Measurements included height, weight, waistline, and blood pressure. Each physical measurement project was performed jointly by two investigators. Height measurement was performed using a TZG height measuring instrument with a range of 2.0 m and a minimum scale of 0.1 cm. The participants used electronic body scales to measure their weight on an empty stomach in the early morning. The scale was accurate to 0.1 kg, with a maximum weight of 150 kg. With the use of a waist ruler with a length of 1.5 m, a width of 1 cm, and a minimum scale of 0.1 cm, the participants measured their waist circumference on an empty stomach in the early morning, with the horizontal position of the lower edge of the axillary costal arch and the midpoint of the line connecting the anterior superior iliac crest as the measuring point. Blood pressure was measured using OMRON HEM-7071 or HEM-770A electronic sphygmomanometer, accurate to 1 mmHg. The participants undertook the first measurement after sitting for 5 min, and after completing one measurement, they loosened the armband. The next measurement was performed after 1 min of sitting for a total of three times, and the average value of the last two measurements is the final blood pressure value.

### Index definition and grouping

BMI was calculated by dividing weight in kilograms by height in meters squared. According to the Chinese national standards, underweight was defined as a BMI of less than 18.5; normal weight, 18.5 to 23.9; overweight, 24.0 to 27.9; and obesity, 28.0 or higher [11]. Abdominal obesity was defined as a waist circumference of 85 cm or greater for men and 80 cm or greater for women [12]. According to the physical activity metabolic equivalent (MET) value of the physical activity outline put forward by Ainsworth [13], physical activity was divided into high, medium, and low intensity, and the corresponding MET values were 8.0, 4.0, and 3.3, respectively. The amount of physical activity per week was equal to the sum of the MET of various intensities multiplied by the weekly activity time (min/week). Physical activity of less than 600 MET-min/week was defined as physical inactivity. According to the World Health Organization recommendations, an average daily intake of less than 400 g of fruits and vegetables was defined as insufficient intake. According to the recommendations of the World Cancer Research Fund, an average daily intake of red meat greater than 100 g was defined as excessive intake.

### Outcomes

The outcomes of this study were IGT and IFG. *Diagnostic criteria of IGT and IFG*: According to the standard established by the WHO [14], IGT participants were classified as participants who did not have diabetes mellitus but who had a fasting plasma glucose (FPG) level of 7.0 mmol/L or lower and a 2-h post-load plasma glucose level of 7.8–11.1 mmol/L. IFG participants were classified as participants who did not have diabetes mellitus but had an FPG level of 6.1–7.0 mmol/L and a 2-h post-load plasma glucose level of 7.8 mmol/L or greater.

### Statistical analyses

We used SAS 9.4 software for data collection and analysis. For missing variables, such as unknown age, the ID card number was used to determine the birth date, and the birth date was subtracted from the survey date to obtain the age. For unknown gender, we used the second-to-last digit of the ID card number, with the odd number representing male and the even number representing female. For continuous variables, we used mean ± standard deviation to describe normal distribution or approximate normal distribution, and used *t* test or *F* test for the comparison between groups. For variables that are not normally distributed, they were described by *M*(P_25_, P_75_). We used the Wilcoxon rank-sum test or Kruskal–Wallis rank-sum test for intergroup comparison. For the classified data, we used the number of cases and constituent ratio n (%) and used the chi-square test for intergroup comparison. For the management of lost to follow-up, the characteristics of the participants who were lost to follow-up were compared with those who were followed up. We used the unconditional logistic regression model for univariate and multivariate analyses, and used a BMI of 18.5–23.9 kg/m^2^ as the reference to calculate the odds ratio (*OR*) value and 95% confidence interval (95% CI) of BMI grouping and outcome. The selection of variables included in the model is first based on the data of this study, through single factor analysis to understand the influencing factors of IGT and IFG, and then combined with literature review and professional knowledge to make it clear that this study needs to include the mixed factors of BMI, IGT, and IFG model and then classify these factors into the model (the specific variables included are shown in Table 2).

## Results

### Baseline characteristics

There were a total of 5,578 participants from the CCDRFS. There were significant differences in the distribution of age, location, smoking, family history of diabetes mellitus, hypertension, dyslipidemia, abdominal obesity, FPG, and 2-h plasma glucose among different BMI groups (*P* < 0.05) (Table 1).

**Table 1:** Baseline characteristics of research participants

Characteristic	Total (*n* = 5,578)	BMI (kg/m^2^)			
		< 18.5 (*n* = 232)	18.5–23.9 (*n* = 2,856)	24.0–27.9 (*n* = 1,770)	≥28.0 (*n* = 720)
Age, n (%)					
18–	2,625 (47.1)	139 (59.9)	1,424 (49.8)	741 (41.9)	321 (44.6)
45–	2,035 (36.5)	48 (20.7)	948 (33.2)	748 (42.2)	291 (40.4)
60–	918 (16.4)	45 (19.4)	484 (17.0)	281 (15.9)	108 (15.0)
Men, n (%)	2,391 (42.9)	103 (44.4)	1,266 (44.3)	735 (41.5)	287 (39.9)
Education, n (%)					
Illiterate	547 (9.8)	21 (9.1)	274 (9.6)	174 (9.8)	78 (10.8)
Primary school	1,793 (32.2)	76 (32.8)	940 (32.9)	557 (31.5)	220 (30.6)
Junior school	1,926 (34.5)	85 (36.6)	986 (34.5)	596 (33.7)	259 (36.0)
Senior high school and above	1,312 (23.5)	50 (21.5)	656 (23.0)	443 (25.0)	163 (22.6)
Total household income^a^, n (%)					
< 10,000	990 (19.7)	40 (18.9)	520 (20.0)	300 (18.8)	130 (20.8)
10,000–	1,290 (25.6)	54 (25.5)	655 (25.2)	415 (26.1)	166 (26.6)
20,000–	1,469 (29.2)	59 (27.8)	763 (29.3)	475 (29.8)	172 (27.5)
36,000–	1,282 (25.5)	59 (27.8)	664 (25.5)	402 (25.3)	157 (25.1)
Urban, n (%)	2,519 (45.2)	107 (46.1)	1,253 (43.9)	860 (48.6)	299 (41.5)
Smoking, n (%)	1,596 (28.6)	74 (31.9)	898 (31.4)	449 (25.4)	175 (24.3)
Drinking, n (%)	2,171 (38.9)	90 (38.8)	1,159 (40.6)	662 (37.4)	260 (36.1)
Family history of diabetes mellitus^b^, n (%)	293 (5.9)	2 (1.0)	132 (5.3)	100 (6.3)	59 (9.1)
Hypertension, n (%)	1,996 (35.8)	37 (16.0)	730 (25.6)	797 (45.0)	432 (60.0)
Dyslipidemia, n (%)	2,776 (49.8)	84 (36.2)	1,173 (41.1)	1,029 (58.1)	490 (68.1)
Abdominal obesity, n (%)	2,389 (42.8)	6 (2.6)	455 (15.9)	1,251 (70.7)	677 (94.0)
Insufficient intake of fruits and vegetables, n (%)	5,327 (95.5)	223 (96.1)	2,728 (95.5)	1,687 (95.3)	689 (95.7)
Excessive intake of red meat, n (%)	1,893 (33.9)	83 (35.8)	1,043 (36.5)	575 (32.5)	192 (26.7)
Lack of physical activities, n (%)	833 (14.9)	37 (15.9)	403 (14.1)	285 (16.1)	108 (15.0)
FPG, mmol/L	5.1 (4.7, 5.5)	5.0 (4.5, 5.4)	5.1 (4.7, 5.5)	5.2 (4.8, 5.6)	5.3 (4.9, 5.6)
2-h plasma glucose, mmol/L	5.5 (4.7, 6.3)	5.1 (4.4, 5.8)	5.3 (4.6, 6.1)	5.6 (4.9, 6.4)	5.8 (5.1, 6.6)

*Note*. *BMI* body mass index, *FPG* fasting plasma glucose

^a^Lack of cases = 547

^b^Lack of cases = 634

During an average follow-up of 6.4 years, 562 developed IGT and 257 developed IFG. There were no participants with both IFG and IGT in this study. The incidence of IGT in patients with normal weight, underweight, overweight, and obesity was 12.9/1,000, 17.0/1,000, 19.0/1,000, and 22.8/1,000 person-years, respectively. Moreover, the incidence of IFG in patients with normal weight, underweight, overweight, and obesity was 6.0/1,000, 2.8/1,000, 8.4/1,000, and 13.5/1,000 person-years, respectively. After age, gender, location, physical activity, family history of diabetes mellitus, hypertension, abdominal obesity, and dyslipidemia were adjusted, overweight increased the risk of IGT by 35% (*OR* 1.35, 95% *CI* 1.08–1.70), and obesity increased the risk of IGT by 77% (*OR* 1.77, 95% *CI* 1.27–1.47). After the factors consistent with the above were adjusted, only obesity increased the risk of IFG by 122% (*OR* 2.22, 95% *CI* 1.39–3.54) (Table 2). When BMI was used as a continuous variable, BMI was linearly and positively correlated with the risk of IGT and IFG. After regression analysis, a 1 kg/m^2^ higher level of BMI was associated with approximately 3% (*OR* 1.03, 95% *CI* 1.00–1.07) higher risk of IGT and approximately 8% (*OR* 1.08, 95% *CI* 1.04–1.13) higher risk of IFG.

**Table 2:** Relationship between BMI and the risk of IGT and IFG

Outcome	BMI (kg/m^2^)				Pearson^2^	*P*
	18.5–23.9 (*n* = 2,856)	< 18.5	24.0–27.9	≥ 28.0		
		(*n* = 232)	(*n* = 1,770)	(*n* = 720)		
IGT						
Number of events	227	24	203	98		
Follow-up year	17,633.2	1,413.1	10,709.3	4,297.5		
Incidence rate (no./1,000 person-years)	12.9	17.0	19.0	22.8		
Odds ratio (95% CI)						
Model 1 [AIC = 3,409.76]	1.00 (Ref)	1.34 (0.86, 2.11)	1.55 (1.27, 1.90)	2.12 (1.64, 2.74)	24.93	0.096
Model 2 [AIC = 3,389.97]	1.00 (Ref)	1.33 (0.85, 2.09)	1.51 (1.23, 1.85)	2.12 (1.64, 2.75)	314.55	0.196
Model 3 [AIC = 3,378.10]	1.00 (Ref)	1.38 (0.88, 2.17)	1.35 (1.08, 1.70)	1.77 (1.27, 2.47)	1,116.15	0.618
IFG						
Number of events	105	4	90	58		
Follow-up year	17,633.2	1,413.1	10,709.3	4,297.5		
Incidence rate (no./1,000 person-years)	6.0	2.8	8.4	13.5		
Odds ratio (95% CI)						
Model 1^*^ [AIC = 1,961.46]	1.00 (Ref)	0.48 (0.18, 1.33)	1.49 (1.11, 1.99)	2.69 (1.92, 3.77)	20.06	0.271
Model 2^*^ [AIC = 1,763.82]	1.00 (Ref)	0.58 (0.21, 1.61)	1.57 (1.15, 2.14)	3.00 (2.11, 4.26)	212.43	0.167
Model 3^*^ [AIC = 1,759.32]	1.00 (Ref)	0.61 (0.22, 1.69)	1.33 (0.94, 1.88)	2.22 (1.39, 3.54)	559.29	0.441

*Note*. Model 1 adjusts age and gender. Model 2 adjusts urban and rural areas, physical activity, and education on the basis of Model 1. Model 3 adjusts hypertension, abdominal obesity, and dyslipidemia on the basis of Model 2. Model 1^*^ adjusts age and gender. Model 2^*^ adjusts urban and rural areas, physical activity, and family history of diabetes mellitus on the basis of Model 1. Model 3^*^ adjusts hypertension, abdominal obesity, and dyslipidemia on the basis of Model 2^*^

*BMI* body mass index, *IGT* impaired glucose tolerance, *IFG* impaired fasting glucose

### Subgroup analysis

Taking the normal weight as a reference, in IGT outcome, only smoking was found to modify the relationship between obesity and IGT (interaction *P* < 0.05). Taking the normal weight as a reference, in IFG outcome, only age was found to modify the relationship between obesity and IFG (interaction *P* < 0.05) (Fig. S1). The analysis results of other subgroups are shown in Table S1.

## Discussions

The purpose of this study was to investigate the relationship between BMI and risk of IGT and IFG and to explore the effect of obesity on the incidence of IGT and IFG in Chinese adults. In China, the incidence of IGT is higher than that of IFG. Compared with the normal weight, the risk of IGT and IFG in obese people in this study is indeed much higher, and the risk of IGT is also increased in overweight people, but there appears to be no statistically significant association between IFG and overweight people. The subgroup analysis indicated that we should also focus on smokers in the prevention of IGT and people older than 45 years in the prevention of IFG.

In our study, the incidence of IGT is higher than that of IFG. A review investigated whether there were differences in the prevalence of prediabetes phenotype among different ethnic groups and concluded that the prevalence of IGT is higher in Asian populations [15]. The possible reason is that Chinese eating habits, unlike Western eating habits which are characterized by high fat content and high glycemic index carbohydrate load, and another reason may be the lack of exercise (such as a sedentary lifestyle) [15]. Moreover, compared with IFG, IGT has a higher risk and is more likely to develop into diabetes [16, 17]. Thus, we should pay more attention to the prevention and control of the IGT population.

Obesity is a major condition of insulin resistance [18]. In East Asia, small weight gain leads to insulin resistance and an increased risk of diabetes [19, 20]. At the same time, obesity is an important determinant of the stronger association between insulin resistance and diabetes in Chinese adults [21]. Studies have shown that a reasonable diet and moderate physical activity can reduce the incidence of prediabetes and diabetes [22, 23]. Moreover, weight loss caused by lifestyle changes can reduce the risk of prediabetes by approximately 50% [24]. At present, to curb the progression of type 2 diabetes, healthcare policies now advocate lifestyle adjustments for high-risk groups, which usually focus on losing weight through behaviors such as diet and increased physical activity [25]. However, because of the characteristics of the current lifestyle and the prolonged sedentary time during study and most work [26], the rates of obese people in China are still intensifying [27]. The results of the CCDRFS showed that the prevalence of obesity had reached 16.5% in 2018 [3]. Therefore, China should actively advocate and increase the intensity of weight control, which can be achieved through diet and exercise, with severe cases requiring surgical treatment.

In this study, obesity is a common risk factor for IGT and IFG, which is consistent with the conclusions of many studies [28, 29]. However, in the overweight stage, the risk of IGT has increased, but the impact on the incidence of IFG is not statistically significant. The possible reason is that there are great differences in potential pathophysiology between IFG and IGT. In the Inter99 study, it was found that IFG was characterized by dysfunction of insulin secretion, followed by decreased insulin sensitivity in the liver, whereas IGT was related to the decrease of systemic insulin sensitivity and then the decrease of β-cell function [30]. Compared with participants with IGT and IGT plus IFG, those with IFG may not necessarily benefit from current lifestyle changes in preventing type 2 diabetes mellitus [31]. This difference may occur because the Diabetes Prevention Program lifestyle changes target pathophysiological mechanisms that are not prominent in IFG [32]. However, a 2019 study reported similar clinical and metabolic responses to lifestyle interventions in people with different plasma glucose states. Moreover, it reported that lifestyle intervention had no effect on fasting plasma glucose levels and 2-h post-load plasma glucose levels in patients with IFG, whereas both indexes decreased in patients with IGT [33]. Moreover, the Whitehall II observational cohort study showed that as plasma glucose returns to normal levels, the expected cardiovascular risk reduction applies only to patients with IGT, not to patients with IFG [34]. However, studies have shown that the 2-h plasma glucose monitoring standard after taking 75 g of glucose is generally not used for screening [35], failing to recognize the presence of IGT. In addition, using fasting plasma glucose alone to screen Asian people missed 3/4 of participants with IGT [36]. Therefore, when screening in the future, we should monitor not only fasting plasma glucose for convenience but also plasma glucose 2 h after taking 75 g of glucose. In addition, for overweight people, it is necessary to strengthen the intervention for IGT patients to eliminate the barriers to prediabetes and diabetes prevention. Moreover, according to the results of the subgroup analysis, attention should be focused on the screening of IGT patients who are smokers and IFG patients who are older than 45 years.

The strengths of our study include its representative sample of Chinese adults, the classification of obesity on the basis of Chinese national standards, and the large sample size, which contributed to the strong statistical support of the adjusted logistic regression analysis. In addition, we analyzed the association between different phenotypes of prediabetes and obesity. According to the different characteristics, the corresponding prevention suggestions and measures were put forward to provide data support for the government's prevention and control of prediabetes and diabetes. However, several limitations of this study deserve to be mentioned. Although most of the confounding factors were considered in this study, because of the large number of prediabetes factors, the baseline survey did not collect all the exposure information, and some unmeasured indicators (such as hormone levels, anxiety, and depression) were not included in the model adjustment, which may have led to residual confusion. These limitations provide a direction for the study of the incidence and influencing factors of prediabetes in the future.

In conclusion, our results show that obesity is an important risk factor for IGT and IFG, and that the risk of IGT will increase in overweight populations in China. The Chinese government should strengthen the prevention and control of obesity and even overweight to reduce the incidence of prediabetes and diabetes.

## Supplementary Information

**Additional file 1:** **Fig. S1  **Subgroup analysis of theassociation between BMI and the risk of IGT and IFG. **Table S1.** Subgroup analysis of the association between BMI and the risk of IGT and IFG

## Data Availability

The datasets used and analyzed during the current study available from the corresponding author on reasonable request.
