# Supplementary material for: Relationship between BMI and risk of impaired glucose tolerance and impaired fasting glucose in Chinese adults: a prospective study
Source: BMC Public Health. 2023 Jan 3;23:14. doi: 10.1186/s12889-022-14912-0 (PMC9811686; doi:10.1186/s12889-022-14912-0)
Supplement: Supplementary file 1 — Additional file 1: Fig. S1 Subgroup analysis of theassociation between BMI and the risk of IGT and IFG. Table S1. Subgroup analysis of the association between BMI and the risk of IGT and IFG [file 12889_2022_14912_MOESM1_ESM.docx]

**Fig. S1** Subgroup analysis of the association between BMI and the risk of IGT and IFG


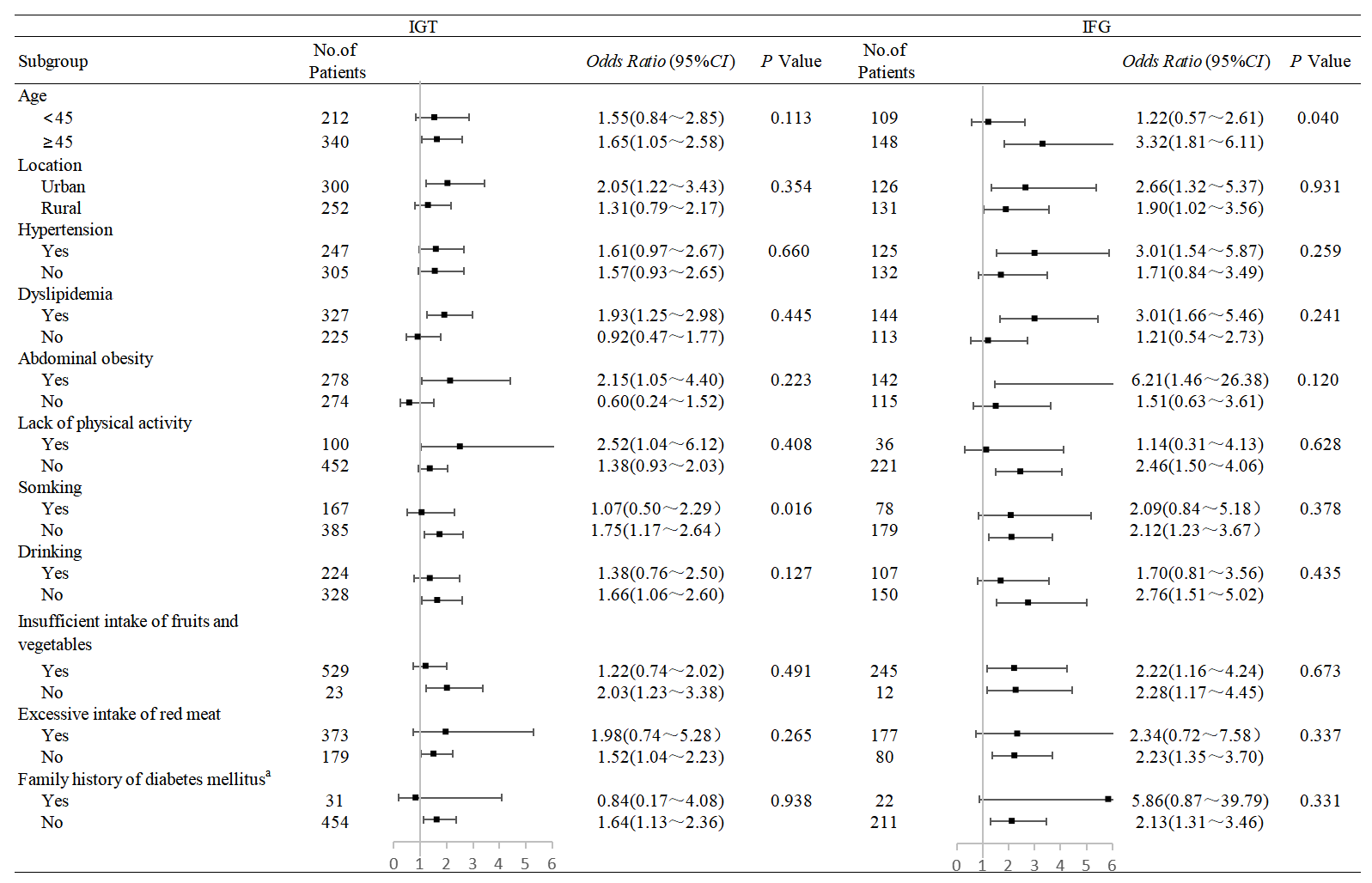


Report the risk effect values of IGT and IFG in obese people with reference to normal BMI; model adjusts age, location, hypertension, dyslipidemia, abdominal obesity, lack of physical activity, smoking, drinking, insufficient intake of fruits and vegetables, excessive intake of red meat and family history of diabetes mellitus, excluding stratification factors; ^a^lack of cases=634

| TableS1 Subgroup analysis of the association between BMI and the risk of IGT and IFG | | | | | | | | | | | | |
| --- | --- | --- | --- | --- | --- | --- | --- | --- | --- | --- | --- | --- |
| Variable | Cases of IGT | BMI(kg/m2)[OR(95%CI)] | | | | *P* Value | Cases of IFG | BMI(kg/m2)[OR(95%CI)] | | | | *P* Value |
|  |  | 18.5～24  (n=2856) | ＜18.5  (n=232) | 24～28  (n=1770) | ＞28  (n=720) |  |  | 18.5～24  (n=2856) | ＜18.5  (n=232) | 24～28  (n=1770) | ＞28  (n=720) |  |
|  |  |  |  |  |  |  |  |  |  |  |  |  |
| Age |  |  |  |  |  | 0.113 |  |  |  |  |  | 0.040 |
| ＜45 | 212 | 1.00(Ref) | 1.30(0.71-2.40) | 1.29(0.93-1.78) | 1.55(0.84-2.85) |  | 109 | 1.00(Ref) | 0.23(0.03-1.67) | 0.99(0.59-1.68) | 1.22(0.57-2.61) |  |
| ≥45 | 340 | 1.00(Ref) | 1.59(0.72-3.49) | 1.49(1.02-2.18) | 1.65(1.05-2.58) |  | 148 | 1.00(Ref) | 1.38(0.41-4.63) | 1.68(1.05-2.69) | 3.32(1.81-6.11) |  |
| Location |  |  |  |  |  | 0.354 |  |  |  |  |  | 0.931 |
| Urban | 300 | 1.00(Ref) | 1.46(0.76-2.80) | 1.36(0.98-1.89) | 2.05(1.22-3.43) |  | 126 | 1.00(Ref) | 0.92(0.28-3.05) | 1.74(1.08-2.80) | 2.66(1.32-5.37) |  |
| Rural | 252 | 1.00(Ref) | 1.26(0.61-2.61) | 1.36(0.94-1.98) | 1.31(0.79-2.17) |  | 131 | 1.00(Ref) | 0.31(0.04-2.28) | 1.01(0.61-1.68) | 1.90(1.02-3.56) |  |
| Hypertension |  |  |  |  |  | 0.660 |  |  |  |  |  | 0.259 |
| Yes | 247 | 1.00(Ref) | 1.48(0.49-4.49) | 1.41(0.96-2.08) | 1.61(0.97-2.67) |  | 125 | 1.00(Ref) | 2.13(0.47-9.72) | 1.83（1.05-3.17） | 3.01(1.54-5.87) |  |
| No | 305 | 1.00(Ref) | 1.36(0.79-2.32) | 1.31(0.95-1.81) | 1.57(0.93-2.65) |  | 132 | 1.00(Ref) | 0.35(0.08-1.43) | 1.12(0.70-1.78) | 1.71(0.84-3.49) |  |
| Dyslipidemia |  |  |  |  |  | 0.445 |  |  |  |  |  | 0.241 |
| Yes | 327 | 1.00(Ref) | 1.47(0.72-3.02) | 1.29(0.94-1.79) | 1.93(1.25-2.98) |  | 144 | 1.00(Ref) | NA | 1.39(0.86-2.23) | 3.01(1.66-5.46) |  |
| No | 225 | 1.00(Ref) | 1.33(0.69-2.58) | 1.41(0.97-2.06) | 0.92(0.47-1.77) |  | 113 | 1.00(Ref) | 0.92(0.32-2.59) | 1.33(0.81-2.20) | 1.21(0.54-2.73) |  |
| Abdominal obesity |  |  |  |  |  | 0.223 |  |  |  |  |  | 0.120 |
| Yes | 278 | 1.00(Ref) | NA | 1.35(0.66-2.76) | 2.15(1.05-4.40) |  | 142 | 1.00(Ref) | NA | 3.41(0.80-14.52) | 6.21(1.46-26.38) |  |
| No | 274 | 1.00(Ref) | 1.57(0.98-2.52) | 1.33(1.03-1.73) | 0.60(0.24-1.52) |  | 115 | 1.00(Ref) | 0.60(0.22-1.66) | 1.27(0.87-1.86) | 1.51(0.63-3.61) |  |
| Lack of physical activities |  |  |  |  |  | 0.408 |  |  |  |  |  | 0.628 |
| Yes | 100 | 1.00(Ref) | 1.37(0.72-2.62) | 1.37(0.98-1.91） | 2.52(1.04-6.12) |  | 36 | 1.00(Ref) | 1.07(0.13-8.91) | 0.85(0.32-2.27) | 1.14(0.31-4.13) |  |
| No | 452 | 1.00(Ref) | 1.43(0.69-2.96） | 1.35(0.94-1.94) | 1.38(0.93-2.03) |  | 221 | 1.00(Ref) | 0.54(0.17-1.74) | 1.43(0.99-2.07) | 2.46(1.50-4.06) |  |
| Smoking |  |  |  |  |  | 0.016 |  |  |  |  |  | 0.378 |
| Yes | 167 | 1.00(Ref) | 1.16(0.50-2.69） | 1.09（0.67-1.72） | 1.07(0.50-2.29） |  | 78 | 1.00(Ref) | 0.93(0.21-4.07) | 1.24(0.63-2.47） | 2.09(0.84-5.18） |  |
| No | 385 | 1.00(Ref) | 1.55(0.86-2.80) | 1.52（1.14-2.04） | 1.75(1.17-2.64） |  | 179 | 1.00(Ref) | 0.47(0.11-1.94) | 1.32(0.89-1.97） | 2.12(1.23-3.67） |  |
| Drinking |  |  |  |  |  | 0.127 |  |  |  |  |  | 0.435 |
| Yes | 224 | 1.00(Ref) | 1.16(0.53-2.52) | 1.21(0.83-1.76) | 1.38(0.76-2.50) |  | 107 | 1.00(Ref) | 0.37(0.05-2.73) | 1.44(0.83-2.50) | 1.70(0.81-3.56) |  |
| No | 328 | 1.00(Ref) | 1.60(0.86-2.96) | 1.48(1.07-2.05) | 1.66(1.06-2.60) |  | 150 | 1.00(Ref) | 0.77(0.24-2.52) | 1.24(0.79-1.93) | 2.76(1.51-5.02) |  |
| Insufficient intake of fruits and vegetables |  |  |  |  |  | 0.491 |  |  |  |  |  | 0.673 |
| Yes | 529 | 1.00(Ref) | 0.89(0.40-2.00) | 1.33(0.94-1.88) | 1.22(0.74-2.02) |  | 245 | 1.00(Ref) | 0.51(0.12-2.16) | 1.46(0.90-2.36) | 2.22(1.16-4.24) |  |
| No | 23 | 1.00(Ref) | 1.98(1.07-3.66） | 1.40(0.99-1.99） | 2.03(1.23-3.38) |  | 12 | 1.00(Ref) | 0.73(0.17-3.09) | 1.24(0.76-2.03) | 2.28(1.17-4.45) |  |
| Excessive intake of red meat |  |  |  |  |  | 0.265 |  |  |  |  |  | 0.337 |
| Yes | 373 | 1.00(Ref) | 0.47(0.11-2.06） | 1.02(0.57-1.81） | 1.98(0.74-5.28） |  | 177 | 1.00(Ref) | 0.89(0.11-7.10) | 1.33(0.56-3.20） | 2.34(0.72-7.58） |  |
| No | 179 | 1.00(Ref) | 1.72(1.03-2.88) | 1.47(1.12-1.93) | 1.52(1.04-2.23) |  | 80 | 1.00(Ref) | 0.55(0.17-1.78) | 1.34(0.92-1.96) | 2.23(1.35-3.70) |  |
| Family history of diabetes mellitus^a^ |  |  |  |  |  | 0.938 |  |  |  |  |  | 0.331 |
| Yes | 31 | 1.00(Ref) | 13.31(0.68-260.45) | 1.95(0.61-6.23) | 0.84(0.17-4.08) |  | 22 | 1.00(Ref) | NA | 4.19(1.08-16.31) | 5.88(0.87-39.79) |  |
| No | 454 | 1.00(Ref) | 1.33(0.81-2.16) | 1.34(1.04-1.72) | 1.64(1.13-2.36) |  | 211 | 1.00(Ref) | 0.59(0.22-1.64) | 1.22(0.85-1.75) | 2.13(1.31-3.46) |  |

^a^lack of cases=634
